# Normal variation of clinical mobility of the mandibular symphysis in cats

**DOI:** 10.3389/fvets.2024.1338623

**Published:** 2024-02-19

**Authors:** Sergio Minei, Edoardo Auriemma, Serena Bonacini, Michael S. Kent, Margherita Gracis

**Affiliations:** ^1^Istituto Veterinario di Novara AniCura, Department of Dentistry, Oral and Maxillofacial Surgery, Novara, Italy; ^2^Clinica Veterinaria San Siro AniCura, Department of Dentistry, Oral and Maxillofacial Surgery, Milan, Italy; ^3^Department of Diagnostic Imaging, Istituto Veterinario di Novara AniCura, Novara, Italy; ^4^Dentistry, Oral, and Maxillofacial Surgery Service, School of Veterinary Medicine, William R. Pritchard Veterinary Medical Teaching Hospital, University of California, Davis, Davis, CA, United States; ^5^Center for Companion Animal Health, Department of Surgical and Radiological Sciences, School of Veterinary Medicine, University of California, Davis, Davis, CA, United States

**Keywords:** mandible, symphysis, mandibular symphyseal morphology, mandibular symphyseal mobility, intraoral radiography, cats

## Abstract

**Introduction:**

The primary objective of this retrospective study was to document the normal variation of clinical mobility of the mandibular symphysis in cats and possible associations with bodyweight, age, sex, sexual status, breed and skull morphology. Secondarily, the radiographic appearance of the mandibular symphysis and possible associations with the analyzed data were evaluated.

**Materials and methods:**

Two hundred and sixteen cats of 15 different breeds that underwent maxillofacial, oral and dental procedures from April 2015 to December 2021 were included. Clinical mobility was evaluated under general anesthesia using a 0 to 3 scale in lateromedial (LM) and dorsoventral (DV) directions. The symphysis was radiographically classified on the occlusal radiographic view of the rostral mandibles as fused or open, and with parallel or divergent margins.

**Results:**

Bodyweight ranged from 2.2 to 12.5 kg (median 4.0 kg), age from 4 months to 17 years and 4 months (median 6 years and 4 months). At the first evaluation DV symphyseal mobility was 0 in 177 cases (82%), 1 in 32 cases (14.8%) and 2 in 7 cases (3.2%), LM mobility was 0 in 61 cases (28.3%), 1 in 110 cases (50.9%) and 2 in 45 cases (20.8%). 81.1% of the radiographs were included in the statistical analysis. Three symphyses (1.6%) were classified as fused and 190 (98.4%) as open, 129 (68.8%) having divergent margins and 61 (31.6%) parallel. One hundred and forty-eight cases (76.7%) did not show the presence of odontoclastic replacement resorption on the canine teeth (TR subgroup 1), 23 (11.9%) showed stage ≤3 lesions (TR subgroup 2) and 22 (11.4%) stage 4 lesions (TR subgroup 3). Logistic regression models exploring factors that affected DV and LM mobility were statistically significant (*p* < 0.0001; *p* < 0.0001) with an increase in LM mobility predicting an increase in DV mobility, and vice versa. An increase in DV mobility was associated with an increase in age and in having resorptive lesions. A decrease in LM symphyseal mobility was associated with being brachycephalic.

**Conclusion:**

The great majority of cases showed some degree of LM symphyseal mobility, and 18% showed DV mobility. Symphyseal bony fusion is rare but possible.

## 1 Introduction

Similarly to dogs (1), the cat’s mandibular symphysis is described as a fibrocartilaginous joint, or synchondrosis, joining the rostral portion of right and left mandibles (2–4). It is reported to be associated with a relatively high degree of mobility and a radiographically appreciable radiolucent gap interposed between the symphyseal plates (5–7). Most small carnivores show a cuneiform symphyseal fibrocartilaginous pad (5). However, the pad of *Felis catus* is slightly asymmetrical and irregular (5). The cuneiform pad has been described as bigger and more solid as compared to the irregular pad, functioning as a core on which the plates articulate (5, 8). Also, great variation in symphyseal mobility within the different species of the Felidae family, including the domestic cat, has been observed, with evidence sometimes of a stiffer symphysis that microscopically or radiographically may appear partially or fully fused (5, 6, 9). A flexible symphyseal joint may allow for better alignment of carnassial teeth and a more efficient shearing action during mastication, in cats as well as other carnivores with a unilateral masticatory pattern (5, 8, 10–14). Different conditions and diseases such as trauma, open mouth jaw locking, severe periodontitis, neoplasia, and the presence of odontoclastic resorptive lesions affecting the mandibular rostral teeth can potentially affect symphyseal mobility and integrity, and radiographic appearance (6, 15–22).

The normal degree of symphyseal mobility is still unknown, and very little information is available on normal symphyseal radiographic characteristics. The primary aim of the present study was to document the normal variation of clinical mobility of the mandibular symphysis in cats, and evaluate possible correlations with bodyweight, age, sex, sexual status, breed and skull morphology. Additionally, the radiographic appearance of the mandibular symphysis and possible associations with the aforementioned variables were assessed.

## 2 Materials and methods

All animals included in the study were client-owned cats anesthetized between April 2015 and December 2021 for diagnosis and treatment of oral and/or maxillofacial conditions. Data collected for each animal included signalment (i.e., bodyweight, age, sex, sexual status, breed, and skull morphology), clinical symphyseal mobility, radiopacity and radiographic shape of the mandibular symphysis. To evaluate possible statistical differences with the general population, brachycephalic cats were also analyzed separately. The breeds that were considered brachycephalic included Birman, British shorthair, exotic, Persian, and Scottish fold (23–25). Further statistical analysis involved the evaluation of cases based on body maturity, classifying immature cats as those ≤1 year of age at the time of initial presentation, and mature cats as those >1 year of age. Patients were excluded in cases of recent or previous maxillofacial trauma involving the mandibles (cases with localized, mild maxillary trauma of known origin were included); neoplastic and other diseases that caused severe bone lysis and remodeling involving the mandibles rostral to the molar area; severe periodontal disease (i.e., AVDC stage 4^1^: more than 50% of attachment loss) affecting the mandibular canine teeth; absence of one or both mandibular canine teeth; and advanced odontoclastic replacement resorption (i.e., AVDC type 2 resorptive lesions, stage 5^2^: remnants of dental hard tissue only visible as irregular radiopacities) affecting the mandibular canine teeth. To evaluate possible influences on bone remodeling and symphyseal mobility by odontoclastic replacement resorption affecting the roots of the mandibular canine teeth, the study population was also divided into three subgroups: cats without lesions (tooth resorption or TR subgroup 1), cats with stage ≤3 lesions (i.e., mild to moderate dental hard tissue loss, with most of the tooth retaining its integrity) affecting one or both canine teeth (TR subgroup 2), and cats with stage 4 lesions (i.e., extensive dental hard tissue loss, with most of the tooth having lost its integrity) affecting one or both canine teeth (TR subgroup 3). The subgroups were statistically evaluated separately and finally compared to the whole population.

**Figure 1:**
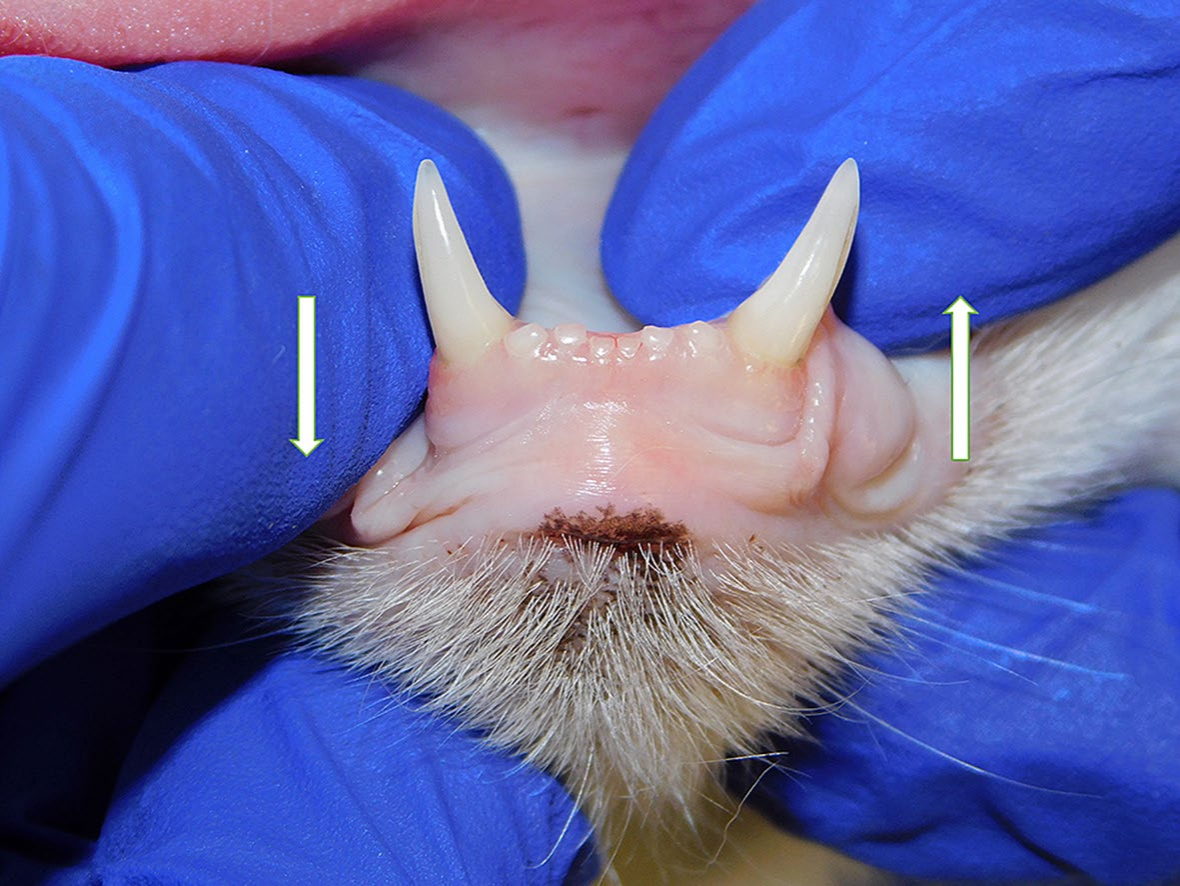
Dorsoventral mobility was evaluated by firmly holding right and left mandibles behind the canine teeth and alternatively pushing in opposite directions (i.e., one mandible in ventral direction and the other one in dorsal direction).

**Figure 2:**
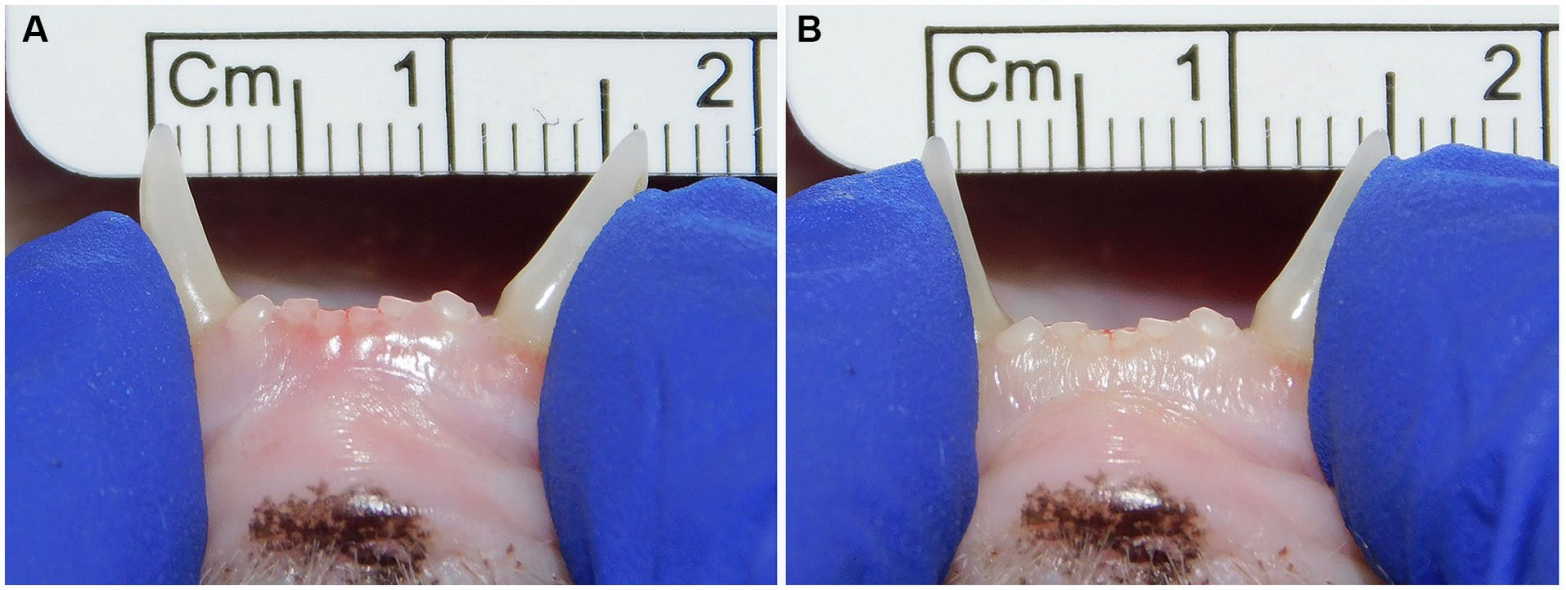
Example of lateromedial mobility evaluation in a clinical case. **(A)** Before applying any force; **(B)** Approximation of the canine teeth by 1 mm (grade 1) after pressing the coronal tips with the thumb and index fingers of the same hand in a lingual direction. The index finger of the free hand is placed on the skin over the ventrocaudal aspect of the symphysis to better appreciate any slight movement.

Symphyseal mobility was evaluated in both the lateromedial (LM) and dorsoventral (DV) directions, as previously described (26). Briefly, during DV mobility evaluation, right and left mandibles were firmly held behind the canine teeth and alternatively pushed in opposite directions (i.e., one mandible in ventral direction and the other one in dorsal direction) (Figure 1). The degree of mobility was recorded as 0 (i.e., no mobility), 1 (i.e., independent movement of the mandibles with a ≤ 1 mm variance at the level of the incisor teeth or the alveolar margin), 2 (i.e., independent movement of the mandibles with a 1–3 mm variance at the level of the incisor teeth or the alveolar margin) or 3 (i.e., independent movement of the mandibles with a > 3 mm variance at the level of the incisor teeth or the alveolar margin). LM mobility was evaluated by pressing the coronal tip of right and left mandibular canine teeth with the thumb and index fingers of the same hand in a lingual direction and visually evaluating any induced movement (i.e., approximation of the tip of the canine teeth) from the front (Figure 2). The placement of a finger of the free hand on the skin over the ventrocaudal aspect of the symphysis further helped determine the presence of slight mobility. A grade 0 to 3 mobility scale was used (i.e., grade 0: no mobility; grade 1: ability to approximate the tip of the canine teeth by ≤1 mm; grade 2: ability to approximate the tip of the canine teeth 1–3 mm; grade 3: ability to approximate the tip of the canine teeth >3 mm). If an animal was examined more than one time, measurements from each visit were recorded and statistically evaluated separately. All clinical evaluations were performed by two operators (MG and SM).

**Figure 3:**
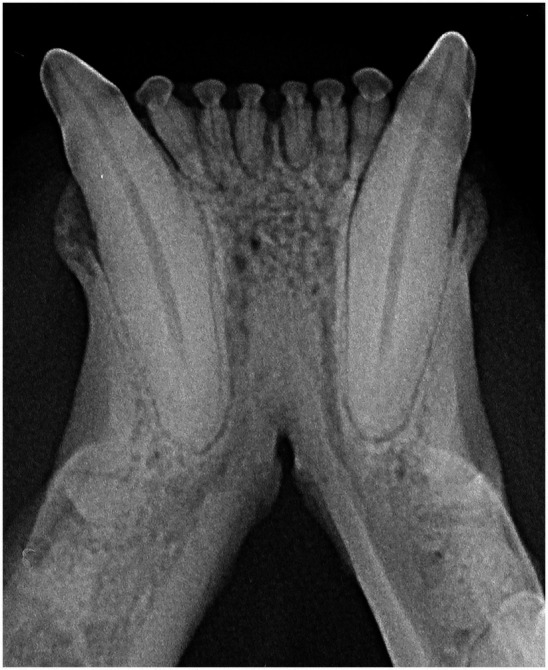
A fused symphysis, in an 8 years and 10 months-old, male castrated, Maine coon cat. Note that the canine teeth were affected by moderate periodontal disease.

**Figure 4:**
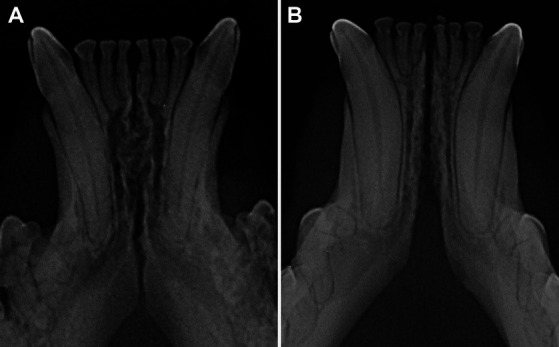
Radiographic appearance of open symphyses, with parallel (**A** 5 years and 11 months-old, domestic shorthair cat) and divergent (**B** 4 years and 2 months-old, sphynx cat) margins.

The radiographic examination of the rostral mandibles and symphyseal area was performed in all cases, but images were excluded from the statistical analysis if radiographic plate positioning, radiographic beam angle, and exposure were considered of insufficient quality for complete evaluation. The examination was performed on a single image obtained using the intraoral, rostrocaudal, bisecting angle technique for the mandibular canine teeth. Instrumentation included a dental radiographic machine (Gendex Oralix AC, Dental Systems, Milan, Italy) and CR (Computed Radiography) digital radiographic plates of variable sizes, based on patient’s size (VistaScan, Dürr Dental SE, Bietigheim-Bissingen, Germany). All DICOM files were saved as JPEG files and stored in a computer (MacBook Pro, Apple Inc., California, USA). Only brightness and contrast were digitally adjusted, if necessary. No other settings were modified. Radiographically, the symphysis was classified as open (i.e., symphyseal plates appearing separated by a radiolucent gap); or fused (i.e., the symphysis appearing as having completely or partially bone radiopacity) (Figure 3). Open symphyses were further described as having parallel margins (i.e., the margins are parallel to each other and to the midline, from the alveolar margin along the entire longitudinal symphyseal extension) (Figure 4A) or divergent margins (i.e., the margins diverge progressively in a rostrocaudal direction) (Figure 4B). A blinded evaluation of the radiographic images was performed independently by four authors (i.e., SM, SB, MG, EA) without information regarding the clinical grading. Cases classified differently by the evaluators were discussed and finally classified over a common consensus.

## 3 Statistical analysis

Data (i.e., date of examination, bodyweight, age, sex, sexual status, breed and skull morphology, number of consults, degree of LM and DV symphyseal mobility, and radiographic appearance) was recorded in a commercially available spreadsheet, and statistical analyses were conducted using a commercially available statistics program (Stata version 14.2, Stata Corporation, College Station Texas, USA). Descriptive statistics were performed to report demographic data. Continuous data was assessed for normality by visualization of distributional plots and use of a Shapiro–Wilk normality test. When continuous data was normally distributed, means and standard deviations were reported; otherwise, medians and overall range were reported. Totals and percentages were used to describe categorical data. Associations between categorical data were evaluated using a Fisher’s exact test. To explore factors that affected mobility in LM or DV directions and the radiologic score, ordered logistic regression was performed. Logistic regression was done to evaluate potential factors affecting radiographic appearance. *P* values <0.05 were considered significant.

## 4 Results

Two hundred and sixteen cats of 15 different breeds were included in the study, accounting for a total of 238 clinical evaluations (Supplementary Table 1). None of the continuous variables was normally distributed. The number of observations per cat varied from 1 in 194 cases (89.8%), to 2 in 18 cases (8.3%), 3 in 3 cases (1.4%) and 4 in 1 case (0.5%). The median period of time between different evaluations was 11.5 months, with a range from 2 weeks to 3 years and 10 months. The elapsed time between evaluations was not standardized.

The most common breeds (i.e., > 9 individuals) were domestic shorthair (*n* = 151, 69.9%), followed by Maine coon (*n* = 24, 9.2%), and British shorthair (*n* = 10, 4.6%). Twenty-three cats were considered to be of brachycephalic breeds including 1 Birman, 10 British shorthair, 2 exotic, 8 Persian and 2 Scottish fold. Bodyweight ranged from 2.2 to 12.5 kg (median 4.0 kg). Age ranged from 4 months to 17 years and 4 months (median 6 years and 4 months). Fourteen cats were ≤ 1 year of age (6.5%) and 202 were > 1 year of age (93.5%). 5.1% of cats were intact males, 52.3% neutered males, 0.9% intact females and 41.7% neutered females.

Radiographs were considered of acceptable quality in 193 (81.1%) cases. Three cases (1.6%) were classified as having a fused symphysis and 190 (98.4%) an open symphysis. The margins of the open symphyses were considered divergent in 129 cases (67.9% of the open symphyses and 66.8% of all cases with evaluable radiographs) and parallel in 61 cases (32.1% of the open symphyses and 31.6% of all cases with evaluable radiographs). There was no significant difference in radiographic appearance over time for the cases that were examined more than once (*p* = 0.49).

A logistic regression model exploring whether any factor (i.e., repeated visits, bodyweight, age, sex, sexual status, breed, skull morphology, DV or LM mobility and the presence of resorptive lesions) affected radiographic appearance (i.e., fused/open, parallel/divergent symphyses) showed no statistical significance (*p* = 0.37). There was also no difference in radiographic appearance between immature and mature cats (*p* = 0.81).

One hundred and forty-eight (76.7%) out of 193 cases with acceptable radiographs did not show evidence of odontoclastic replacement resorption affecting the roots of the canine teeth (TR subgroup 1), 23 cases (11.9%) had stage ≤3 resorptive lesions (TR subgroup 2) and 22 cases (11.4%) stage 4 resorptive lesions (TR subgroup 3).

The degree of DV and LM symphyseal mobility at the time of the first evaluation for the whole population and different subgroups is described in Table 1.

**Table 1:** Symphyseal mobility recorded at the time of the first evaluation.

Total population (216 cats)					
DV mobility score	Number of cases	Percentage (%)	LM mobility score	Number of cases	Percentage (%)
0	177	82	0	61	28.3
1	32	14.8	1	110	50.9
2	7	3.2	2	45	20.8
3	0	0	3	0	0
TR subgroup 1 [148 out of 193 cases with acceptable radiographs (76.7%)]					
0	130	87.8	0	40	27
1	16	10.8	1	79	53.4
2	2	1.4	2	29	19.6
TR subgroup 2 [23 out of 193 cases with acceptable radiographs (11.9%)]					
0	13	56.5	0	3	13
1	7	30.4	1	13	56.5
2	3	13.1	2	7	30.5
TR subgroup 3 [22 out of 193 cases with acceptable radiographs (11.4%)]					
0	15	68.2	0	9	40.9
1	7	31.8	1	8	36.4
2	0	0	2	5	22.7
Brachycephalic breeds (22 cases, 10.2%)					
0	19	86.4	0	11	50
1	3	13.6	1	10	45.5
2	0	0	2	1	4.5
Immature cats (≤1 year) (14 cases, 6.5%)					
0	14	100	0	5	35.7
1	0	0	1	7	50
2	0	0	2	2	14.3
Mature cats (>1 year) (202 cases, 93.5%)					
0	163	80.7	0	56	27.7
1	32	15.8	1	103	51
2	7	3.5	2	43	21.3

**Figure 5:**
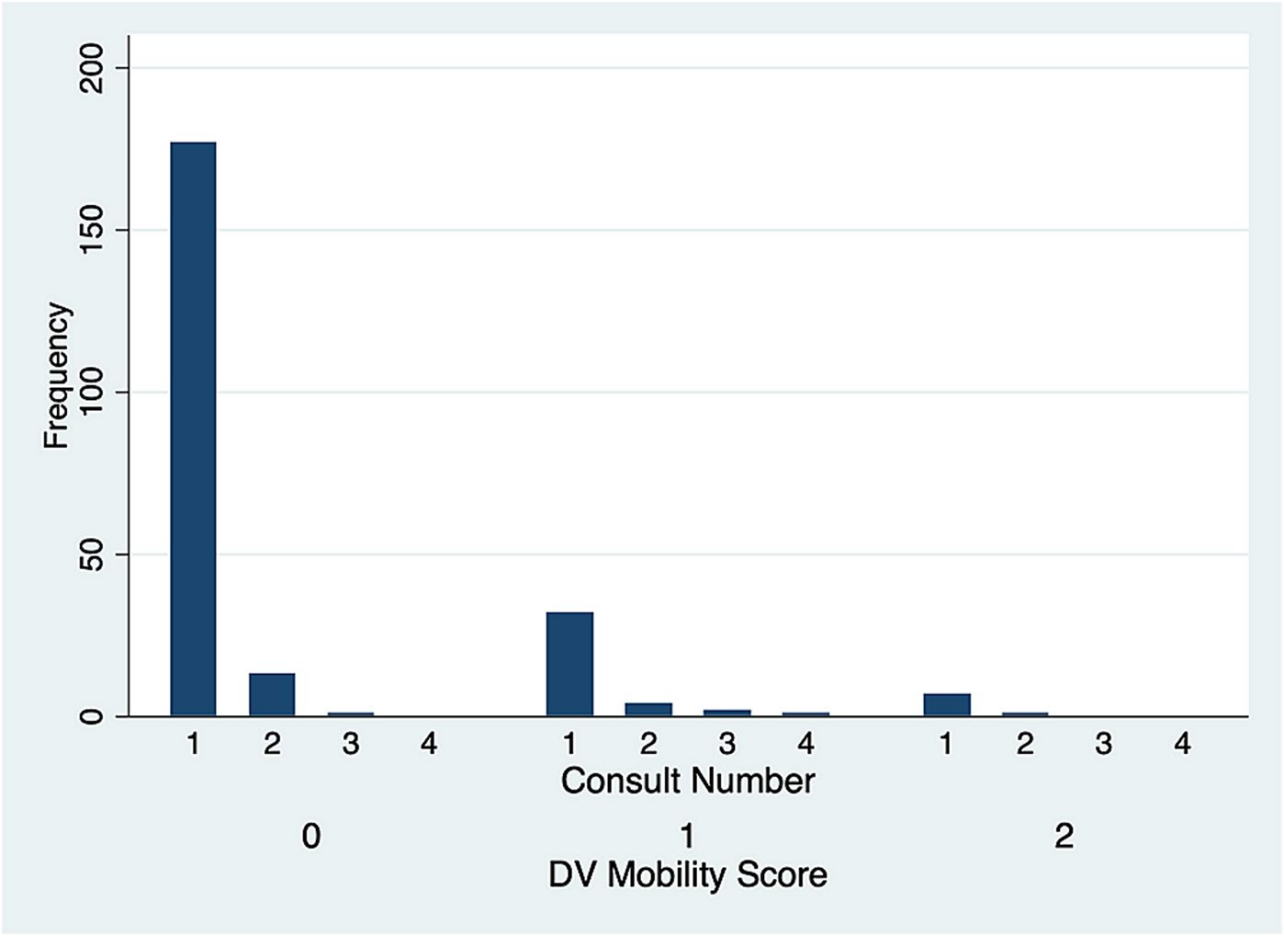
Dorsoventral (DV) symphyseal mobility score frequency over subsequent evaluations.

**Figure 6:**
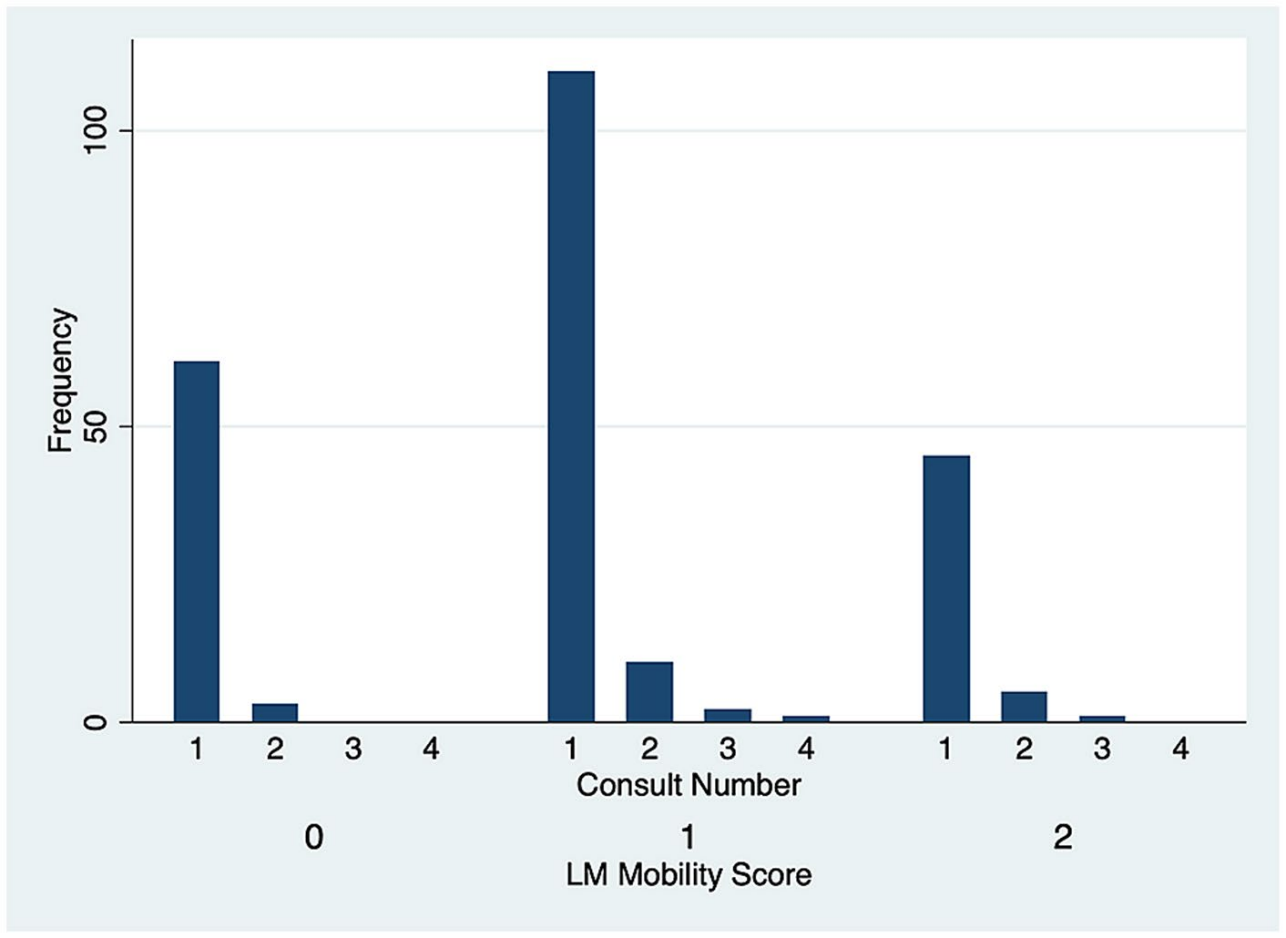
Lateromedial (LM) symphyseal mobility score frequency over subsequent evaluations.

There was no significant difference in DV (*p* = 0.51) (Figure 5) and LM (*p* = 0.80) (Figure 6) mobility scores over time for the 22 cases that were examined more than once.

The DV mobility was statistically different for TR subgroups (*p* < 0.001), with a proportional increase in subgroups 2 and 3 compared to subgroup 1. There was no statistical difference in LM mobility between TR subgroups (*p* = 0.22).

There was no statistical difference in DV mobility scores between brachycephalic and non-brachycephalic cats (*p* = 1.00), and there was also no association between being brachycephalic and an increase in TR score (*p* = 0.80).

There was no statistical difference in DV (*p* = 0.22) or LM (*p* = 0.81) mobility between immature and mature cats, and no significant association between age subgroups and symphyseal mobility or radiographic appearance.

An ordered logistic regression model exploring factors that affected DV symphyseal mobility was statistically significant (*p* < 0.0001), with an increase in DV symphyseal mobility associated with an increase in age (*p* = 0.001, OR 1.02, 95% CI 1.01–1.03) and with an increase in LM mobility (*p* = 0.001, OR 2.61, 95% CI 1.46–4.66). A logistic regression model exploring factors affecting LM symphyseal mobility was also statistically significant (*p* < 0.0001), with an increase in LM mobility being associated with an increase in DV mobility (*p* = 0.001, OR 3.26, 95% CI 1.77–6.01), an increase in bodyweight (*p* = 0.04, OR 1.28, 95% CI 1.01–1.60) and with being a non-brachycephalic breed (*p* = 0.03, OR 0.37, 95% CI 0.15–0.90). There was no statistical difference in mobility scores for animals of different breeds or sex/sexual status in the ordered logistic regression model.

## 5 Discussion

### 5.1 Anatomy, physiology and classification

Most of the available information about the anatomy and morphology of the mandibular symphysis in carnivores derives from observational and histologic studies published by Scapino using anatomical specimens and a museum skull collection (5, 8). Four different classes were described, based on bone and soft tissue morphology (Table 2). The symphysis of domestic cats (*Felis catus*) was classified as class I, a fibrocartilaginous joint composed of right and left symphyseal plates that, as compared to dogs, show a smoother surface or only a few low irregularities that fit loosely into shallow valleys of the opposite side (3, 5).

**Table 2:** Symphyseal classes by Scapino (5).

Symphyseal classes (5)	Symphyseal plates	Symphyseal space	Fibrocartilagineuos pad	Soft tissues	Symphyseal flexibility
Class I	Flat or with few, low interdigitating irregularities (ridges and valleys); a smooth, conspicuous craniodorsal area	Wider caudally than rostrally	Cuneiform shape, wider dorsally than ventrally on transverse section and wider rostrally than caudally in coronal section	Threewalled fibrous capsule, deep dense fibrous ligaments [dorsal transverse, ventral transverse, ventral oblique (external) and internal cruciate (central area)]; a central and aboundant venous plexus, wider caudally than rostrally	Maximum flexibility: basic movements visible to the naked eye and manually easy to produce
Class II	Ridges and valleys more numerous and intimately related than in class I; presence of a smooth craniodorsal area	Narrower as compared to class I, with approximately the same width all along the symphysis	Thinner as compared to class I	Thick and short fibrocartilagineus and fibrous ligament fibers running nearly transversely across the joint; venous plexus less abundant rostrally than caudally	Limited flexibility: visible movements, but manually more difficult to produce
Class III	Plates irregularities taller and interdigitating more that in class II; absent or small smooth craniodorsal area	Narrower space as compared to class II, wth approximately the same width all along the symphysis	Smaller as compared to class II, irregular in shape	Ligaments fibers mostly transverse and caudally irradiated in all directions; reduced or nearly absent venous plexus as compared to class II	Stiff: minute amounts of visible movements under forceful manipulation
Class IV	Bony fusion	Not present	Not present	Absent, with non-lamellar bone obliterating the joint space	Rigid: no visible movements

Scapino also highlighted how the flexibility of carnivores’ mandibular symphysis varied and correlated to its anatomical classification (Table 2) (5). Slightly differently from our suggested technique, mobility was evaluated in lateral and medial directions by turning the tip of each canine tooth in both directions, and in rostrocaudal and dorsoventral directions by attempting to slide one symphyseal plate over the other. The symphysis of domestic cats (*Felis catus*) was determined to have no mobility in craniocaudal and dorsoventral directions but a high degree of flexibility in lateral and medial directions (5). Another more recent, unpublished study by Gawor (22) on 64 clinical cases evaluated symphyseal mobility in the lateromedial direction with the use of an elastic chain applied to the mandibular canine teeth and measuring the tips distance before and 30 s after application. This was a possibly more objective way to evaluate LM mobility than the technique that we used, which entails a certain degree of subjectivity. However, we aimed to find an easy, fast and clinically applicable way of evaluating symphyseal mobility in two directions, which will take only a few seconds to perform by any operator after a very short period of training.

Despite the inherent differences existing when evaluating live animals versus anatomical specimens, the results of the present study are in agreement with the Scapino studies, as we showed that in feline clinical cases there is greater mobility in LM compared to the DV direction, possibly because of tighter dorsal ligaments and dorsal joint capsule as compared to the ventral structures. These findings also overlap those reported in a dog study from our group (26). However, the percentage of cats showing mobility of the symphysis in either direction was much higher than in dogs, with 18% of cats showing some DV mobility and 71.7% of cats showing some LM mobility, as compared to 2.8 and 29.3% of dogs, respectively. It is possible that the increased symphyseal mobility helps during mastication, allowing carnassial teeth alignment and a more effective shearing action, compensating the limited laterolateral mobility at the temporomandibular joint typical of cats (27, 28).

It is surprising that Scapino found significant symphyseal mobility in the mediolateral direction, as the tight dorsal mucoperiosteum would be expected to limit this type of movement. However, we only tested lateromedial mobility, and cannot confirm or challenge these findings. Further studies are warranted to better evaluate any degree of flexibility in other directions.

### 5.2 Radiographic evaluation

In this study radiography was the imaging modality of choice, as it still represents the most commonly used imaging modality for dental patients. However, radiology carries some limitations. When evaluating the radiopacity of the symphyseal space it should always be considered that even a minimal degree of variation in exposure as well as lateral or rostrocaudal angulation of the radiographic beam could modify the overall appearance (i.e., degree of radiolucency vs. radiopacity) (6), and that high quality images are necessary to differentiate between fused and open symphyses. For these reasons, we had to exclude from analysis nearly one fifth of the images. Also, the use of a single projection (i.e., occlusal) likely limited our ability to diagnose the presence of odontoclastic resorptive lesions of the canine teeth. The use of advanced modalities such as computed tomography and cone beam computed tomography could have certainly added further insights into the evaluation of the shape, size, and roughness of the symphysis, and presence and distribution of odontoclastic resorptive lesions.

**Figure 7:**
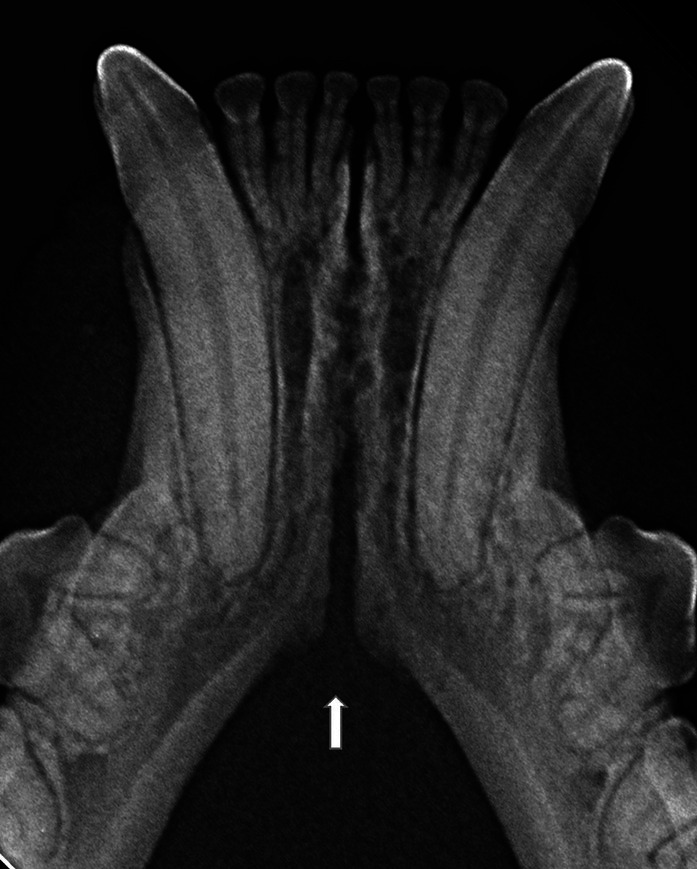
An example of a case with a relatively clear change in angulation of the mandibular ventral cortex (arrow), identified as the caudal extension of the symphysis, in a 2 years and 1 month old, female spayed, mesocephalic domestic shorthair cat.

**Figure 8:**
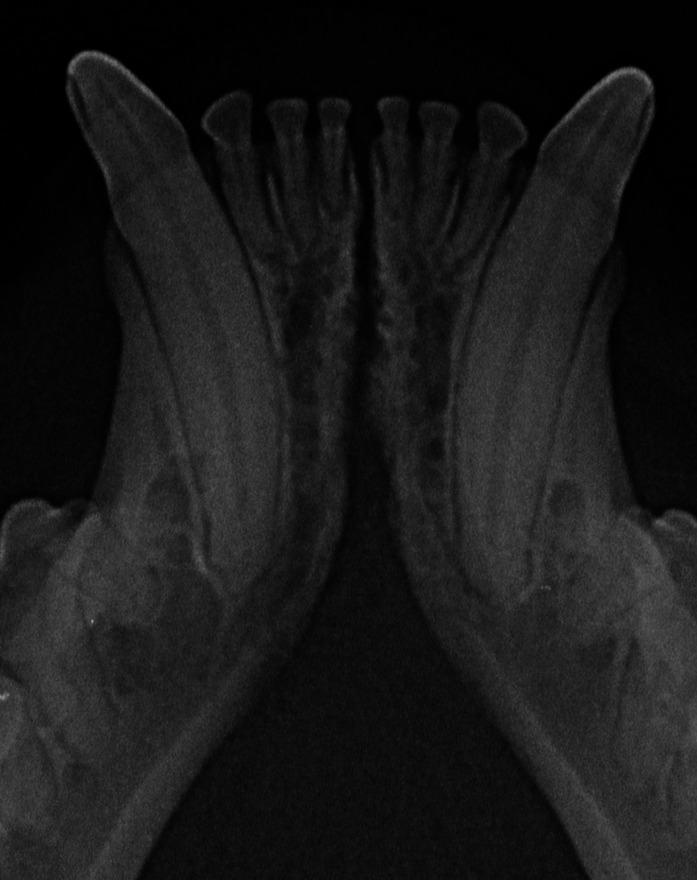
Example of a case with a smooth transition from the symphyseal area to the ventral cortex of the mandibular body, in a 2 years and 6 months-old, male castrated, mesocephalic domestic shorthair cat.

In the present study, as well as in our canine study (26), it was initially attempted to transform the Scapino’s four-class anatomical classification (5) into a similar radiographic classification. However, reaching a common consensus on the degree of radiopacity of the symphyseal space between evaluators was not possible for many of the radiographic images. In addition, determining the caudal symphyseal extension was not always straightforward. In most of the cases, in an area that varied from the area of projection of the canine teeth apex to the area of projection of the third premolar tooth, there was a relatively clear angle of the mandibular ventral cortex, that we identified as the possible caudal extension of the symphysis (Figure 7). However, in some cases this anatomical landmark seemed to be nearly absent, and the symphyseal plates made a smooth transition with the ventral cortex of the mandibular body, without an obvious radiographic difference in shape or angulation (Figure 8). Interestingly, in dogs the sharply angled outline of the distal symphyseal area was not as frequent as in cats, and was mostly seen in brachycephalic breeds (26).

Scapino also described the symphyseal space as being wider caudally than rostrally, both in dogs and in cats (5). In the present study, almost 70% of the cases showed a caudally divergent symphyseal space, and about a third of the cases had radiographically parallel symphyseal plates, which was more similar to Scapino’s class II symphyses (Table 2) (5), showing that the symphysis in cats is more variable in shape than previously reported. We also found a fused symphysis in two Maine coon (Figure 3) and in a single Persian cat. Maine coon and Persian cats were among the most common pure breed cats seen in the study population, and the finding of fused symphyses may simply be a type 1 error. So, no conclusions about a possible breed predisposition to fused symphyses can be made at this stage. Further anatomical and histological studies defining the exact extent of the mandibular symphysis would help better elucidate possible variations of this anatomical area.

### 5.3 Age

The cats’ mandibular symphysis has been reported to undergo some changes with aging (6). The continuous forces applied over time during mastication on the symphysis may cause degradation of the fibrocartilaginous pad and connective tissues, and favor a compensatory osteogenic production from skeletal and soft tissues, possibly leading to a stiffer symphysis (6, 12–14). Indeed, in a recently published canine study by our group it was shown that an increase in age was associated with a decreased mobility (26). In the present study the results were different, with no statistical difference in clinical mobility between immature and mature cats, and a only statistical association between increasing age and an increase in DV symphyseal mobility in the logistic regression model.

**Figure 9:**
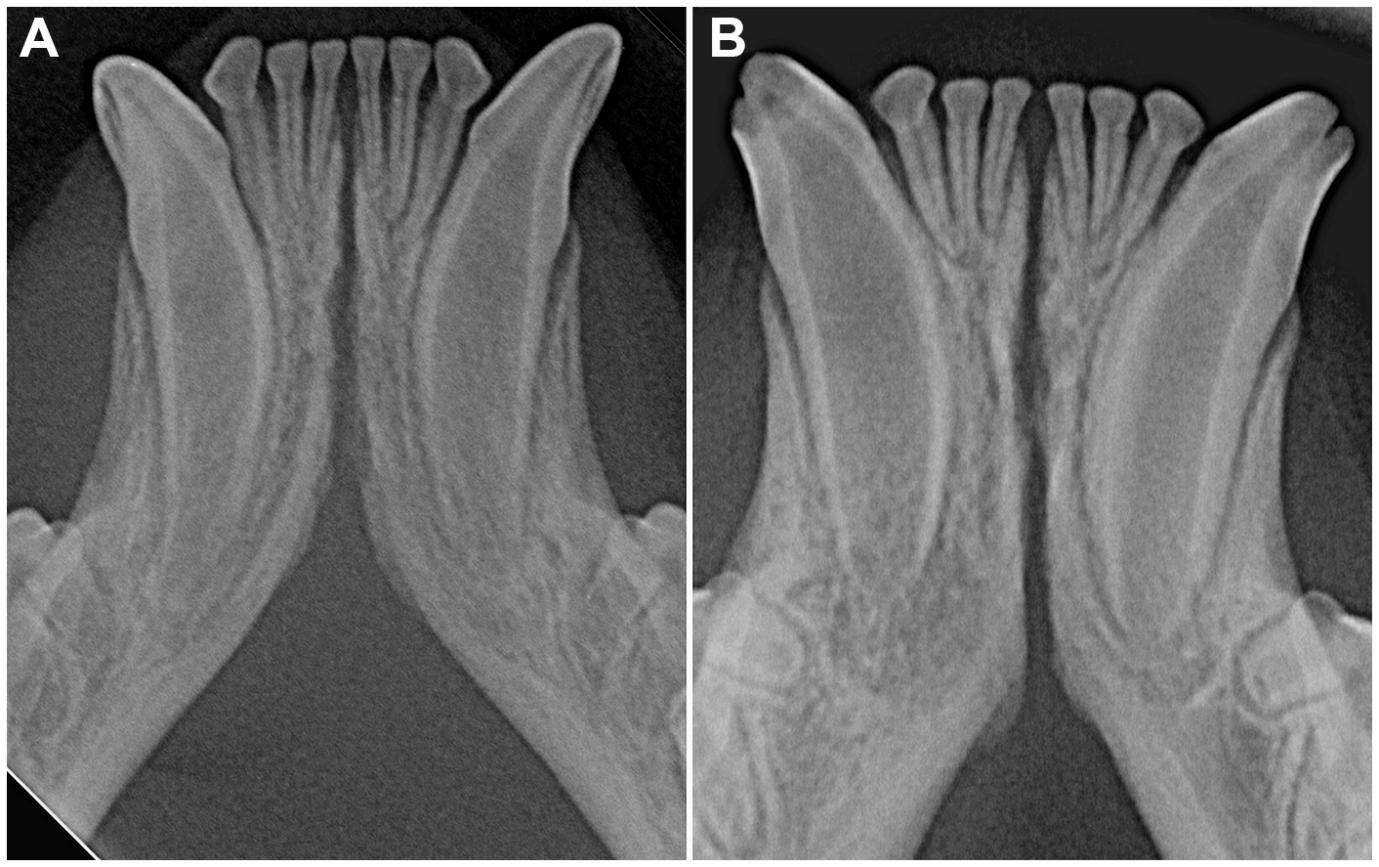
Variation of symphyseal shape in immature cats. **(A)** 6 months-old, Siberian cat with an open, divergent symphysis; **(B)** 7 months-old, Siamese cat with an open, parallel symphyseal plates (note the periapical radiolucency at the right canine tooth due to endodontic disease).

In dogs, the symphysis is more frequently divergent in young dogs and parallel in mature dogs (26). In cats, the symphyseal radiographic shape and width varied within each age subgroup, with parallel and divergent as well as subjectively wide or thin symphyses encountered in all subgroups (Figures 4, 9). It should be considered, though, that only 14 cases were younger than one year, and that these results could be due to a statistical type 1 error.

**Figure 10:**
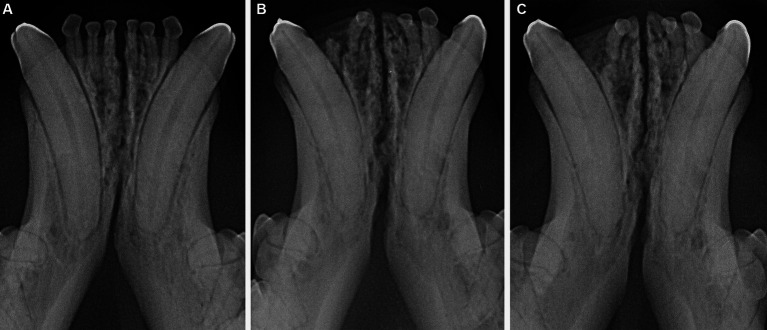
Male castrated, Maine coon cat at 2 years and 2 months **(A)**, 6 years **(B)** and 7 years **(C)** of age, showing lack of significant changes in radiopacity and symphyseal width over time. Mobility was scored as DV = 0 and LM = 1 at the time of the first examination and DV = 1 and LM = 1 at the following evaluations.

No significant changes over time in mobility or radiographic appearance were found in cases that were evaluated more than once (Figure 10). However, again, the number of cases that had multiple evaluations was limited and the time frame between visits varied. Therefore, further analysis in larger populations would need to be performed to confirm these results.

The three cases with a fused symphysis were all mature cats (i.e., cat 1: 7 years and 9 months; cat 2: 8 years and 7 months; cat 3: 9 years and 5 months). They were presented and were treated for variably associated oral/dental diseases not involving the mandibular symphysis, including periodontal disease, missing teeth, tooth resorption, stomatitis, and pyogenic granuloma. No previous trauma was reported in the recent or past history, and no anatomical abnormalities were present. These cases were evaluated only once in their lifetime; therefore, any changes over time cannot be assessed. However, in agreement with the few existing reports (6, 9), even if it is rare, ossification of the mandibular symphysis in cats should be considered possible.

### 5.4 Skull morphology, bodyweight and size

The main factors affecting bite forces in dogs and cats are bodyweight and skull morphology and size (29). In carnivores, a stiffer symphysis could be an adaptation to transfer higher occlusal forces from the balancing to the working side of the mandible (5). However, other studies have shown that fused and unfused symphyses are equally able to transfer dorsally orientated forces with efficacy, as cruciate ligaments and interdigitating plates’ irregularities create sufficient stiffness and resistance in the sagittal plane, being able to resist dorsoventral shearing forces (11). In dogs, bite forces increase in brachycephalic compared to dolichocephalic breeds (29), and the symphysis in brachycephalic canine breeds has been described as having varying degrees of mobility (6, 7), but no details are available in the literature regarding feline species.

In the present study, there was no statistical difference in mobility score or radiographic appearance for any specific breed. This is in contrast to dogs, where being a brachycephalic breed was shown to be associated with a higher mobility in the DV direction (26). Skull morphology also did not seem to affect LM mobility in dogs (26), while being a brachycephalic cat in the present study was associated with a lower LM mobility score, possibly indicating a stiffer symphysis in these cases. However, it should be acknowledged that breeds classification based on skull morphology may vary based on different studies and type of indexes used (23–25). Therefore, our results related to brachycephalic cats should be confirmed in future studies, with more precise skull measurements and classification of patients, using more reproducible criteria.

The association between an increase in LM symphyseal mobility and an increase in bodyweight was significant (*p* = 0.04), possibly indicating the presence of looser ventral symphyseal ligaments in larger sized individuals. These findings are the opposite to those described in dogs, where an increase in bodyweight was associated with an increase in symphyseal stiffness (26). It is not clear why the canine and feline studies disagreed in this regard, but it should be considered that the body size in dogs can vary much more than in cats.

### 5.5 Clinical application

Symphyseal mobility in cats has been reported to be affected by conditions such as trauma, inflammation, tooth resorption, infection, or bone proliferation (6). In particular, maxillofacial trauma often leads to symphyseal separation, which has been shown to develop in up to 73% of trauma cases, alone or in association with other bony lesions (15–17, 30–33). Considering that the symphysis may also be fused, it should also be noted that occasionally a true symphyseal fracture rather than separation may occur.

However, available information on the expected normal degree of symphyseal mobility is scarce (5, 6). The present study showed that some mobility may be expected even in healthy (as defined in the materials and methods section) feline patients, and that a mild to moderate degree of flexibility may not implicate the necessity for treatment. On the other hand, a mobility score of 3 may indicate an abnormal clinical situation, which may warrant further investigation to exclude the presence of disorders affecting this area. This assumption should be confirmed by future studies performed in pathological conditions.

While the main aim of this study was to report the normal variation of symphyseal mobility, cases affected by odontoclastic replacement resorptive lesions were still included, as they are very common in feline patients (34–36). In this population the radiographic prevalence of replacement resorptive lesions affecting the canine teeth was 23.3%, but it should be noted that only the occlusal projection was evaluated, and it is possible that the disease was thus underdiagnosed. In particular, the authors were interested in finding out if bone remodeling accompanying replacement resorptive lesions of the canine teeth could affect symphyseal mobility and radiographic appearance, and if there were any differences associated with different stages of the disease (37–39). It has been reported that the shape, length and radiopacity of the mandibular symphysis can change accordingly with the presence of odontoclastic resorptive lesions, resulting in an absent or narrower symphyseal space with rough surfaces, and increased stiffening (6). However, contrary to this theory in the present study there was no difference in LM mobility between cats with or without resorptive lesions, and an increase in DV mobility in cats with tooth resorption affecting the canine teeth, possibly excluding symphyseal involvement in the described bone remodeling process. The reason for the increased DV mobility in these patients remains unclear, but the presence of eventually painful lesions could reduce masticatory forces applied to the mandibles, possibly leading to ligamentary relaxation at the symphysis.

## 6 Conclusion

The main aims of the present study were to describe and classify the normal clinical mobility and the radiographic appearance of the mandibular symphysis in cats, and to identify any potentially influencing factors. The great majority of cases showed some degree of LM mobility, while only 18% of cats showed some degree of DV mobility. When mobility in one direction was present, some mobility in the other direction was also more likely. A DV and LM mobility score higher than 2 may indicate the presence of disease affecting the symphysis. Similarly to dogs, in cats the mandibular symphysis mostly appeared radiographically open with a divergent shape, with just a few rare cases showing a fused symphysis. There was no statistical difference in mobility scores or radiographic appearance for animals of different breeds or sex/sexual status, or between immature and mature cats. However, the logistic regression model showed that an increase in age and the presence of AVDC type 2 resorptive lesions affecting the canine teeth were associated with an increase in DV symphyseal mobility. On the other hand, LM symphyseal mobility was slightly increased in animals of higher bodyweight, and decreased in brachycephalic cats, as compared to the rest of the study population.

To better characterize symphyseal morphology and improve our proposed classification, further studies using advanced imaging modalities and histological evaluations are warranted. This knowledge will be crucial to better identify the distinction between physiological and pathological conditions, and interpret any variation in the feline mandibular symphysis that could potentially predispose to other diseases.

## Data availability statement

The original contributions presented in the study are included in the article/Supplementary material, further inquiries can be directed to the corresponding author.

## Ethics statement

Ethical approval was not required for the studies involving animals in accordance with the local legislation and institutional requirements because this study is retrospective in nature and included clinical cases; hence, it is exempt from IACUC requirements. Written informed consent was not obtained from the owners for the participation of their animals in this study because the study is retrospective in nature and hence, it is exempt from written informed consent.

## Author contributions

SM: Data curation, Investigation, Methodology, Project administration, Validation, Visualization, Writing – original draft, Writing – review & editing. EA: Data curation, Validation, Visualization, Writing – review & editing. SB: Data curation, Validation, Visualization, Writing – review & editing. MK: Data curation, Formal analysis, Methodology, Validation, Writing – review & editing. MG: Conceptualization, Data curation, Funding acquisition, Investigation, Methodology, Resources, Validation, Visualization, Writing – review & editing.

## Footnotes

^1^American Veterinary Dental College. Nomenclature. Periodontal anatomy and disease. Stages of periodontal disease. Available at: https://avdc.org/avdc-nomenclature/ (Accessed June 25, 2023).

^2^American Veterinary Dental College. Nomenclature. Teeth abnormalities and related procedures. Tooth resorption. Available at: https://avdc.org/avdc-nomenclature/ (Accessed June 25, 2023).
